# Population pharmacokinetics and exposure–response relationships of maribavir in transplant recipients with cytomegalovirus infection

**DOI:** 10.1007/s10928-024-09939-2

**Published:** 2024-09-27

**Authors:** Ivy H. Song, Grace Chen, Siobhan Hayes, Colm Farrell, Claudia Jomphe, Nathalie H. Gosselin, Kefeng Sun

**Affiliations:** 1 Quantitative Clinical Pharmacology, Takeda Development Center Americas, Inc., 500 East Kendall Street, Cambridge, MA 02142 USA; 2 ICON plc, Reading, UK; 3 Certara Drug Development Solutions, Princeton, NJ USA

**Keywords:** Maribavir, Cytomegalovirus, Transplant, Exposure response, Population pharmacokinetics

## Abstract

Maribavir is approved for management of post-transplant cytomegalovirus (CMV) infections refractory and/or resistant to CMV therapies at a dose of 400 mg twice daily (BID). Population pharmacokinetic (PopPK) and exposure–response analyses were conducted to support the appropriateness of 400 mg BID dosing. A PopPK model was developed using non-linear mixed-effects modeling with pooled maribavir plasma concentration–time data from phase 1 and 2 studies (from 100 mg up to 1200 mg as single or repeated doses) and the phase 3 SOLSTICE study (400 mg BID). Exposure–response analyses were performed for efficacy, safety, and viral resistance based on data collected in the SOLSTICE study. Maribavir PK after oral administration was adequately described by a two-compartment model with first-order elimination, first-order absorption, and an absorption lag-time. There was no evidence that maribavir PK was affected by age, sex, race, diarrhea, vomiting, disease characteristics, or concomitant use of histamine H_2_ blockers, or proton pump inhibitors. In the SOLSTICE study, higher maribavir exposure was not associated with increased probability of achieving CMV DNA viremia clearance, nor with reduced probability of treatment-emergent maribavir-resistant CMV mutations. A statistically significant association with maribavir exposure was identified for taste disturbance, fatigue, and treatment-emergent serious adverse events, while transplant type, enrollment region, CMV DNA level at baseline, and/or CMV resistance at baseline were identified as additional risk factors for these safety outcomes. In conclusion, the findings of these PopPK and exposure–response analyses provide further support for the recommended maribavir dose of 400 mg BID.

**Supplementary Information:**

The online version contains supplementary material available at 10.1007/s10928-024-09939-2.

## Introduction

Cytomegalovirus (CMV) infection, although largely asymptomatic in healthy individuals, can have serious implications such as end-organ disease and organ failure in immunocompromised transplant recipients [1, 2]. Anti-CMV therapies such as ganciclovir, valganciclovir, and foscarnet are limited by toxicities and the potential development of resistance [3–5], which may lead to treatment failure [6, 7].

Maribavir (previously known as 1263W94) is an oral benzimidazole riboside with a selective multimodal mechanism of action against human CMV [8, 9] and is indicated for treatment of post-transplant CMV infection and disease that is refractory to prior antiviral treatment [10–12]. In the phase 3 SOLSTICE trial [9], maribavir 400 mg twice daily (BID) demonstrated superior efficacy compared with investigator-assigned therapy (IAT; i.e., valganciclovir/ganciclovir, foscarnet, or cidofovir) in transplant recipients with refractory CMV (with or without resistance). The proportion of patients receiving maribavir or IAT that achieved the primary (confirmed CMV viremia clearance at week 8) and key secondary (CMV viremia clearance and symptom control at the end of week 8, maintained through week 16) endpoints were 55.7% vs. 23.9% (*p* < 0.001), and 18.7% vs. 10.3% (*p* = 0.01), respectively. More patients in the IAT group discontinued study medication due to treatment-emergent adverse events (TEAEs) than in the maribavir group. Dysgeusia (taste disturbance), nausea, vomiting, and diarrhea were commonly experienced TEAEs among patients treated with maribavir in clinical trials [9, 13]. Treatment-emergent (TE) CMV mutations conferring resistance to maribavir have been identified in clinical trials and are consistent with in vitro findings [14–16]. In the SOLSTICE trial, 60 of 234 (25.6%) patients receiving maribavir developed TE maribavir-resistant mutations, while in the AURORA trial 8.8% of maribavir-treated patients developed resistance [13, 15]. The selection of 400 mg BID as the maribavir dose in SOLSTICE [9] was based on two dose-ranging studies [17, 18], and this is the recommended dose of maribavir in product labels [10, 11].

After oral administration, maribavir is rapidly absorbed, with a median post-dose time of 1–3 h to maximum plasma concentration (T_max_) [10, 11, 19]. The pharmacokinetics (PK) of maribavir is dose-proportional after single doses of up to 1600 mg [19] and multiple doses up to 2400 mg per day [10], and maribavir in plasma is approximately 98% bound to plasma proteins [10, 11]. Clearance of maribavir occurs primarily by hepatic metabolism, [20] with renal excretion accounting for < 2% of the total clearance of unchanged drug [19]. Cytochrome P450 (CYP) 3A4 and 1A2 account for 70–85% and 15–30% of CYP-mediated metabolism, respectively, leading to formation of the pharmacologically inactive metabolite VP44469 (previously referred to as 4469W94) [19, 21]. Coadministration with CYP3A4 inhibitors or inducers may therefore affect clearance and plasma concentrations of maribavir [10, 11, 22, 23]. Direct glucuronidation accounts for a small fraction of maribavir metabolism [11, 21]. The mean half-life of maribavir is similar in healthy volunteers (3.0–4.8 h) and transplant recipients (4.3 h) [10, 11, 19]. With a dose of 400 mg BID, steady-state maribavir exposure was reached in transplant recipients with CMV infection within 2 days [10, 11].

To provide further evidence in support of the recommended 400 mg BID daily dose for maribavir, we conducted a population PK (PopPK) analysis using pooled data from multiple phase 1, 2, and 3 studies, and exposure–response analyses based on efficacy, safety, and viral resistance data from the SOLSTICE study. These analyses were also conducted to determine the impact of patient characteristics (e.g., age, sex and race, disease, or health conditions) and extrinsic factors (i.e., concomitant medications) on maribavir PK to determine the potential need for dose adjustment in these populations.

## Methods

### PopPK model development

A PopPK model was developed using non-linear mixed effects modeling (NONMEM) (v7.4.3) with pooled maribavir plasma concentration–time data from 667 participants in nine phase 1 studies (*n* = 182, single or repeated doses of 100, 200 or 400 mg), two phase 2 studies (NCT01611974, EudraCT 2010-024247-32, 400, 800, and 1200 mg BID), and one phase 3 study (SOLSTICE, NCT02931539, 400 mg BID; phase 2 and 3 combined: *n* = 485). The final PopPK analysis dataset comprised data from individuals who had received at least one dose of maribavir and had at least one quantifiable post-dose concentration. This model was used to describe the time course of maribavir plasma concentrations in healthy volunteers, participants with hepatic or renal impairment, stable renal transplant patients, and hematopoietic cell transplant (HCT) or solid organ transplant (SOT) recipients with CMV infection, including those with refractory CMV infection.

One- (ADVAN2 TRANS2), two- (ADVAN4 TRANS4), and three (ADVAN12 TRANS4)-compartment models with first-order absorption, an absorption lag time, and linear clearance were initially investigated. The NONMEM control stream for the final model can be found in the Supplementary Methods S1 of the Supplementary Material 1. From non-compartmental analysis it was known that concomitant administration of ketoconazole (a strong CYP3A4 inhibitor) increased maribavir area under the concentration–time curve from time 0 to infinity (AUC_0-∞_) and maximum plasma concentration (C_max_) by 53% and 10%, respectively [22], and concurrent administration of rifampin (a strong CYP3A4 inducer) significantly reduced plasma concentrations of maribavir, resulting in a 60% reduction in AUC, 30% reduced half-life, and 2.5-fold increased clearance [23]. Consequently, the effects of co-administration of strong CYP3A4 inhibitors and strong CYP3A4 inducers were considered for inclusion in the base model. The effect of body weight (scaled to 70 kg) on both clearance and volume terms was also considered for inclusion in the base model.

Structural and variance model parameters were estimated for the base model, and inter-individual variability (IIV) was included on all structural parameters. Runs with untransformed data were used to test the residual error models. Plots of weighted residuals were evaluated for homoscedasticity with respect to predictions and time since dose, and the structure of the base model was expanded as necessary to best reflect the characteristic shape of the observations over time. Dose and time dependency were explored for PK model parameters. Parameter estimation was performed using Monte Carlo Importance Sampling Expectation Maximization assisted by Mode a Posteriori, with MU referencing (where THETAs that define typical values of individual parameters, and are associated with random effects, are referenced in a PHI/MU format) to improve the efficiency of computations [24, 25]. The impact of post-dose observations below the lower limit of quantification (BLQ) was assessed in initial models by implementing the M3 method, which maximizes the likelihood for all the data treating BLQ observations as censored [26]. The performance of the final PopPK model was evaluated using a confidence interval prediction-corrected visual predictive check (CI-pcVPC) method. In addition, the percentage of observations outside the overall 5th and 95th percentiles of the predicted data was calculated.

### Covariates

The covariates available for evaluation in the PopPK analysis included the following continuous and categorical covariates: age (years), age category (≥ 18 to < 65 years and ≥ 65 to < 80 years), and body mass index (BMI) at baseline; sex; race; health status (healthy, renal impairment, hepatic impairment, transplant recipient with CMV); study; diarrhea; vomiting; dose; disease characteristics (transplant type, baseline plasma CMV DNA, CMV category, hepatic impairment, presence of CMV mutations at baseline, baseline use of antilymphocyte antibody, episode of qualifying CMV infection, prior use of CMV prophylaxis, gastrointestinal [GI] graft-versus-host disease [GvHD]); and drug–drug interactions as categorical covariates (Yes/No) on apparent clearance (histamine H_2_ blockers, proton pump inhibitors [PPI]). Strong CYP3A4 inhibitors and inducers, as well as weight, were included in the base model.

Continuous covariates were obtained from observations on the first day of dosing, or at screening if this value was not available. Available covariates were evaluated and selected for inclusion in the covariate model based on one or more of the following criteria: (1) plots of individual estimates versus covariates demonstrate a correlation where the parameter estimates may increase or decrease with increasing values of a continuous covariate or particular category of a categorical covariate; (2) a statistically significant covariate effect is determined by univariate analysis of variance or by regression analysis (for categorical and continuous covariates, respectively); (3) physiological or pharmacological rationale; and (4) information from prior analyses or published sources. Parameters that showed excessive (> 30%) shrinkage in IIV were carefully reviewed as they can be ill suited for graphical assessment of covariate effects. Categorical covariates were tested and incorporated in the model as a series of index variables taking on values of zero or one.

The full model with backwards deletion approach was used for covariate modeling, with all covariate parameter relationships of interest entered into the model simultaneously. Highly correlated covariates were tested one at a time to avoid confounding in the estimation of covariate effects. A backwards deletion was carried out at the *p* = 0.001 significance level. The order of removal was based on the relative standard error and 95% confidence interval (CI) of the parameter estimate. Covariate-specific parameter estimates were compared and estimates that appeared comparable were combined and tested for significance.

### Individual predicted PK parameters

The final model was used to derive individual estimates of steady-state AUC over one dosing interval (AUC_0–τ_), C_max.ss_, and minimum maribavir plasma concentration (C_trough.ss_) for a 400 mg BID dosing regimen for all participants in the PopPK analysis. Individual estimates of C_max.ss_, C_trough.ss_, and AUC_0–τ_ were obtained by prediction of the concentration–time profiles (concentrations predicted at 0, 1, 2, 3, 4, 6, 8 and 12 h) after a steady-state dose of 400 mg BID for respective individuals using their individual post hoc parameter values in the absence of strong CYP3A4 inducers or strong CYP3A4 inhibitors, and zero values for residual variability. The PK parameters AUC_0–τ_ (linear up/log down trapezoidal rule), C_max.ss_, and C_trough.ss_ were determined by non-compartmental methods.

The mean (percentage coefficient of variation [CV%]), geometric mean (GM) CV%, median, and percentiles for AUC_0–τ_, C_max.ss_, and C_trough.ss_ were obtained overall and by health status (healthy individuals, all transplant recipients with CMV infection, and transplant recipients with refractory CMV infection in the phase 3 SOLSTICE study). For transplant patients with CMV infections, summary statistics were provided by covariates of interest including age (18 to < 65 years and ≥ 65 years; 18 to < 65 years, 65 to < 75 years, and 75 to < 85 years), sex, race, ethnicity, body mass index (BMI; underweight [< 18.5 kg/m^2^], normal weight [18.5 to < 25 kg/m^2^], overweight [25 to < 30 kg/m^2^], obese [≥ 30 kg/m^2^]), concurrent PPI, concurrent H_2_ inhibitors, transplant type, baseline plasma CMV DNA (low and intermediate/high), CMV category, hepatic impairment (none, Child-Pugh Class A, Child-Pugh Class B), presence of CMV mutations at baseline, and baseline use of antilymphocyte antibody. For the PPI and H_2_ inhibitor analyses, PK parameters were computed only for patients receiving the relevant concomitant medication for all their PK observations and for those who never received the relevant concomitant medication. For healthy individuals, summary statistics were provided by the following covariates of interest: age (≤ 65 years, > 65 years), sex, race, ethnicity, and BMI.

### Exposure–response analyses

The exposure–response analyses for maribavir were based on data collected from transplant recipients with CMV infections that were refractory to treatment with ganciclovir, valganciclovir, foscarnet, or cidofovir in the phase 3 SOLSTICE study (ClinicalTrials.gov: NCT02931539) [9]. SOLSTICE was a multicenter, open-label, active-controlled study in which patients were randomized 2:1 to receive maribavir 400 mg BID or IAT for 8 weeks, with 12 weeks of follow-up. The primary endpoint was confirmed CMV clearance at the end of week 8, and the key secondary endpoint was achievement of CMV clearance and symptom control at the end of week 8, maintained through week 16. TE CMV mutation conferring resistance to maribavir was assessed as an exploratory endpoint. Full details of the SOLSTICE study have been published previously [9, 15].

Posterior Bayes parameters of the final PopPK model were used to derive rich concentration–time profiles for maribavir. Actual dosing history and factors affecting the PK of maribavir (i.e., body weight and time-varying presence of strong CYP3A4 inhibitors or strong CYP3A4 inducers) were considered in the predictions. Model-derived exposure parameters of maribavir were calculated from the individual concentration–time profiles: AUC from 0 to 24 h on the day of an adverse event (AE) (AUC_day_); C_max_ of maribavir on the day of an AE (C_max.day_); average concentration of maribavir on each study day (C_avg_); AUC from 0 to 24 h at steady state on the last day of exposure (AUC_ss_); and C_max.ss_ and C_trough.ss_ on the last day of exposure.

Data set preparation, exploration, visualization of the data and exposure-response analyses were performed using R^®^ (version 4.0.5 for efficacy and resistance analyses; version 4.0.2 for safety analysis) with comprehensive R^®^ archive network (CRAN) and Certara Strategic Consulting (CSC) package. Firth correction on logistic regression was done based on library “logistf”. Logistic regressions with log-linear exposure effect or Emax exposure effect were performed using NONMEM Version VII (version 7.4).

#### Exposure–response analysis of efficacy and treatment-emergent mutations

Maribavir exposure parameters (AUC_ss_, C_max.ss_, and C_trough.ss_) were combined with the primary and key secondary efficacy endpoints, and with TE CMV mutations conferring resistance to maribavir from the SOLSTICE study. The following covariates were included in the dataset to identify potential influencing factors: transplant type (HCT, SOT); CMV DNA level (high, intermediate, low) at baseline; symptom status (symptomatic or asymptomatic) at baseline; CMV resistant at baseline (yes, no); donor (D) and recipient (R) CMV serostatus (D+/R−, D+/R+, or D−/R+, D−/R−); antilymphocyte use (yes, no); immune function status as measured by total white blood cells from hematology panel at baseline; CMV-specific CD4 + CD69 + and CD8 + CD69 + T cells from immune function assay as continuous variables at baseline; enrolling region (North America, Europe, Asia Pacific); prior use of CMV prophylaxis (yes, no); age (as a continuous parameter) and age group (12 to < 18 years; 18 to < 45 years; 45 to < 65 years; ≥65 years); sex (male, female); race and ethnicity; TE CMV maribavir-resistant mutations (yes, no); and temporal identification of maribavir resistance (no, on maribavir treatment, post-maribavir treatment).

The exposure–response analyses were performed using a logistic regression model whereby a response can be defined as 0 (non-responder) or 1 (responder). Logistic regression analyses were performed to explore potential associations with maribavir exposure and the probability of response. The logistic regression model had the following form, assuming that maribavir exposure is included in the model:

1 $$\:Ln\:\left(odds\right)=Ln\:\left(\frac{{P}_{i}}{1-{P}_{i}\:}\right)=\:\alpha\:\:+\:\left({\beta\:}_{1}{{\bullet\:}X}_{1}\right)$$

P_i_ is the probability of the event in the ith patient and α is the baseline Ln (odds) of the event. The model assumes a linear exposure effect on logit scale, with the parameter α representing the intercept, while β_1_ represents the slope linking the exposure parameter (X_1_) of maribavir (AUC_ss_, C_max.ss_, and C_trough.ss_) to the response. The statistical significance of α and β_1_ was tested in the logistic regression models with a p value of < 0.05, and 95% CIs were provided for each parameter. The odds ratio (OR) for β_1_ was derived for a specific unit increment of the PK parameter.

The impact of each covariate on responders was explored in the above logistic regression equation using a stepwise approach by integrating relevant covariates one by one. At each step, the covariate retained was the one with the lower Akaike information criterion (AIC) and with statistically significant effect (*p* < 0.05 on β). The logistic regression models including drug exposure and multiple covariates have the following form:

2 $$\:Ln\:\left(odds\right)=Ln\:\left(\frac{{P}_{i}}{1-{P}_{i}\:}\right)=\:\alpha\:\:+\:{\beta\:}_{1}{X}_{1}+{\beta\:}_{2}{X}_{2}+\dots\:{\beta\:}_{n}{X}_{n}$$

P_i_ is the probability of the event in the ith patient and α is the baseline Ln (odds) of the event, and β_1_…β_n_ is the adjusted OR characterizing the dependence of the Ln (odds) on one or more covariates (X_1_…X_n_). For continuous covariates, ORs were derived for a specific increment. For categorical covariates, ORs were derived for the test vs. reference group (e.g., female vs. male). No interactions between covariates were evaluated in the covariate analysis. The Firth correction was implemented in the logistic regression to reduce the bias of maximum likelihood estimates when a separation problem arises in small samples with unbalanced risk factors [27].

Additional logistic regression models were tested:

Log-linear exposure effect:

3 $$\:Ln\:\left(odds\right)=Ln\:\left(\frac{{P}_{i}}{1-{P}_{i}\:}\right)=\:\alpha\:+\:\left({\beta\:}_{1}{{\bullet\:}\text{l}\text{n}(X}_{1}\right))$$

Maximum effect (E_max_) exposure effect [28]:

4 $$\:Ln\:\left(odds\right)=Ln\:\left(\frac{{P}_{i}}{1-{P}_{i}\:}\right)=\:\alpha\:\:+\:\frac{{E}_{max}\times\:{X}_{1}}{{EC}_{50}+{X}_{1}}$$

α is the baseline Ln (odds), EC_50_ is the drug concentration associated with 50% maximal response, and E_max_ is the maximum response.

#### Exposure–response analysis of safety

Descriptive statistics were derived for TEAEs, TE serious AEs (SAEs), and TEAEs of special interest during the 8-week treatment phase. Based on the descriptive statistics results, key TEAEs were selected for a formal exposure–response analysis, and exposure parameters of maribavir (AUC_day_, C_max.day_, C_avg_, AUC_ss_, and C_max.ss_) derived from the final PopPK models were merged with retained TEAE. Logistic regression models were developed to link maribavir exposure to the above probability of TEAEs with the exposure metric resulting in the best-fitting model according to AIC selected to perform the covariate analysis. The statistical significance of covariates was tested for potential risk factors in addition to maribavir exposure in the logistic regression models with a p value of < 0.05, as in the exposure–response analysis of efficacy.

## Results

### PopPK analysis

#### Description of observed data and patient characteristics

Across nine phase 1, two phase 2, and one phase 3 studies, 6163 maribavir concentration records were obtained from 670 enrolled participants. A total of 213 concentrations were BLQ, and 3 individuals only had BLQ concentrations. Consequently, 5950 quantifiable maribavir concentration records from 667 individuals were included in the final PopPK model, including 2378 records from 485 transplant recipients with CMV infection (including refractory CMV infection), 2952 records from 133 healthy volunteers, 148 records from 10 patients with mild or moderate hepatic impairment, 220 records from 20 stable renal transplant recipients, and 252 records from 19 patients with renal impairment (mild, moderate, or severe). Based on M3 analysis, BLQ concentrations had no impact on parameter estimates or model predictions. Patient characteristics are shown in Table 1.

**Table 1:** Summary of patient characteristics in the population pharmacokinetic analysis

Covariates^a^	Phase 1 (*n* = 182)	Phase 2/3 (*n* = 485)	Overall (*n* = 667)
Age category			
18 to < 65 years	174 (96)	374 (77)	548 (82)
65 to < 80 years	8 (4)	111 (23)	119 (18)
Weight (kg)			
Mean (SD)	79.0 (15.3)	74.1 (18.2)	75.4 (17.6)
Median (range)	76.0 (49.1–141)	72.7 (36.1–131)	74.0 (36.1–141)
Sex			
Male	100 (55)	294 (61)	394 (59)
Female	82 (45)	191 (39)	273 (41)
Race			
Caucasian	120 (66)	394 (81)	514 (77)
Black	59 (32)	53 (11)	112 (17)
Asian	1 (1)	16 (3)	17 (3)
Other	2 (1)	22 (5)	24 (4)
Health status			
Transplant with CMV	0	485 (100)	485 (73)
Hepatic impairment	10 (5)	0	10 (1)
Healthy	133 (73)	0	133 (20)
Renal impairment	19 (10)	0	19 (3)
Stable renal transplant	20 (11)	0	20 (3)
Hepatic impairment			
Child-Pugh Class A	0	18 (4)	18 (3)
Child-Pugh Class B	10 (5)	8 (2)	18 (3)
Missing^b^	0	117 (24)	117 (18)
No chronic liver disease	172 (95)	342 (71)	514 (77)
CMV category			
No infection	182 (100)	0 (0)	182 (27)
Asymptomatic infection	0	405 (84)	405 (61)
CMV organ disease	0	36 (7)	36 (5)
Symptomatic infection	0	44 (9)	44 (7)
Presence of CMV mutations			
No – phase 1 studies	182 (100)	0	182 (27)
No	0	261 (54)	261 (39)
Yes	0	206 (42)	206 (31)
Missing	0	18 (4)	18 (3)
Transplant type at baseline			
None	162 (89)	0	162 (24)
Solid organ transplant	20 (11)	284 (59)	304 (46)
Hematopoietic cell transplant	0	201 (41)	201 (30)
Diarrhea			
No	181 (99)	479 (99)	660 (99)
Mild	3 (2)	29 (6)	32 (5)
Moderate	0	15 (3)	15 (2)
Severe	0	3 (1)	3 (0)
Vomiting			
No	182 (100)	474 (98)	656 (98)
Mild	0	31 (6)	31 (5)
Moderate	0	12 (2)	12 (2)
Severe	0	2 (0)	2 (0)
Maximal	0	1 (0)	1 (0)
GI GvHD^c^			
No	182 (100)	481 (99)	663 (99)
Mild	0	7 (1)	7 (1)
Moderate	0	4 (1)	4 (1)
Severe	0	1 (0)	1 (0)
Proton pump inhibitors^c^			
No	178 (98)	161 (33)	339 (51)
Yes	4 (2)	356 (73)	360 (54)
Histamine H_2_ blockers^c^			
No	179 (98)	421 (87)	600 (90)
Yes	3 (2)	77 (16)	80 (12)

CMV, cytomegalovirus; GI GvHD, gastrointestinal graft-versus-host disease; SD, standard deviation

^a^ All data are n (%) unless otherwise stated

^b^ Not collected in phase 2 study (EUDRACT 2010–024247-32)

^c^ Time-varying covariate, so the same participant may be both No and Yes. As a result, the apparent number of participants exceeds 667

#### Final PopPK model description and parameter estimates

The PK of maribavir after oral administration was adequately described in the study populations by a two-compartment disposition model with first-order elimination, first-order absorption, and an absorption lag-time. The model included strong CYP3A4 inhibitor and inducer effects on apparent total clearance (CL/F), dose effect on first-order absorption rate (Ka), and effect of transplant patients with CMV on CL/F. The initially estimated weight exponents on CL/F and apparent volume of peripheral compartment (Vp/F) were deemed imprecise [29], so in the final model the weight effects were fixed rather than estimated. CL/F, apparent volume of central compartment (Vc/F), inter-compartment clearance between central and peripheral compartments (Q/F), and Vp/F all increased with weight fixed to allometric scalars. No other covariate relationship was identified.

The parameter estimates for the final PopPK model are shown in Table 2. CL/F was estimated to be 30% lower in the presence of strong CYP3A4 inhibitors and 2.24-fold higher in the presence of strong CYP3A4 inducers, and to be 24% lower for transplant recipients with CMV infection than for all other individuals. The estimates of IIV were small to moderate (CV ranged from 34 to 53%) for all parameters apart from Q/F, Vp/F, and Ka (91–152%). While the shrinkage of the individual random effects was moderate for Vc/F, Q/F, Vp/F, Ka and lag-time (21–47%), it was low for CL/F (6%). Consequently, individual estimates of AUC_0-τ_ are reliable with low eta-shrinkage for CL/F (6%) but C_max.ss_ may not be as reliable with moderate etashrinkage for Vc/F (21%). When shrinkage is higher than about 20–30%, individual parameter values approach the typical parameter value [30].

**Table 2:** Parameter estimates of the final population pharmacokinetic model

Parameter	NONMEM estimates		
	Estimate^a^	%RSE^b^	Inter-individual variability CV_TVP_^c^ (%RSE)
CL/F (L/h)	3.77	3.79	52.5 (6.43)
Vc/F (L)	18.6	3.45	34.0 (14.4)
Q/F (L/h)	0.908	12.9	90.7 (24.5)
Vp/F (L)	8.66	10.4	103 (20.7)
Ka (h^–1^)	0.336	10.9	152 (14.2)
Lag-time (h)	0.271	5.91	44.1 (28.8)
CL/F ~ weight	0.75 fixed	–	–
Vc/F ~ weight	1 fixed	–	–
Q/F ~ weight	0.75 fixed	–	–
Vp/F ~ weight	1 fixed	–	–
CL/F ~ CYP3A strong inhibitors	0.700	1.98	
CL/F ~ CYP3A strong inducers	2.24	2.95	
Ka ~ dose	– 1.94	6.49	
CL/F ~ transplant patients with CMV	0.756	4.63	
	Estimate^a^	%RSE^b^	Intra-individual variability CV^d^
σ^2^_prop phase 1_	0.0673	2.86	25.9
σ^2^_prop phases 2 & 3_	0.137	3.67	37.0

The reference population was a 70-kg individual without CMV administered 800 mg maribavir in the absence of CYP3A strong inhibitors or inducers. The shrinkages of individual random effects were estimated as 6% for CL/F, 21% for Vc/F, 39% for Q/F, 46% for Vp/F, 35% for Ka and 47% for lag-time

CL/F, apparent total clearance; CV, coefficient of variation; CV_TVP_, typical population value for coefficient of variation; h, hour; Ka, first-order absorption; Q/F, inter-compartment clearance between central and peripheral compartments; RSE, relative standard error; σ2_prop_, proportional residual error; Vc/F, apparent volume of central compartment; Vp/F, apparent volume of peripheral compartment

^a^ Back-transformed from natural log scale, except for σ^2^, CL/F ~ weight, Vc/F ~ weight, Q/F ~ weight, Vp/F ~ weight, CL/F ~ CYP3A strong inhibitors, CL/F ~ CYP3A strong inducers, and Ka ~ dose

^b^ For CL/F (including CL/F ~ transplant patients with CMV), Vc/F, Q/F, Vp/F, Ka, and lag-time, RSE = SE.100. For σ^2^, CL/F ~ weight, Vc/F ~ weight, Q/F ~ weight, Vp/F ~ weight, CL/F ~ CYP3A strong inhibitors, CL/F ~ CYP3A strong inducers, and Ka ~ dose, RSE = SE(θ)/θ.100

^c^$$\:{CV}_{TVP}=\sqrt{{\omega\:}_{P}^{2}}.100$$ if $$\:{\omega\:}_{P}^{2}\le\:0.15$$, else $$\:{CV}_{TVP}=\sqrt{{e}^{{\omega\:}_{P}^{2}}-1}.100$$

^d^ $$\:CV=\sqrt{{{\sigma\:}}^{2}}.100$$

#### Final PopPK model evaluation

**Fig. 1:**
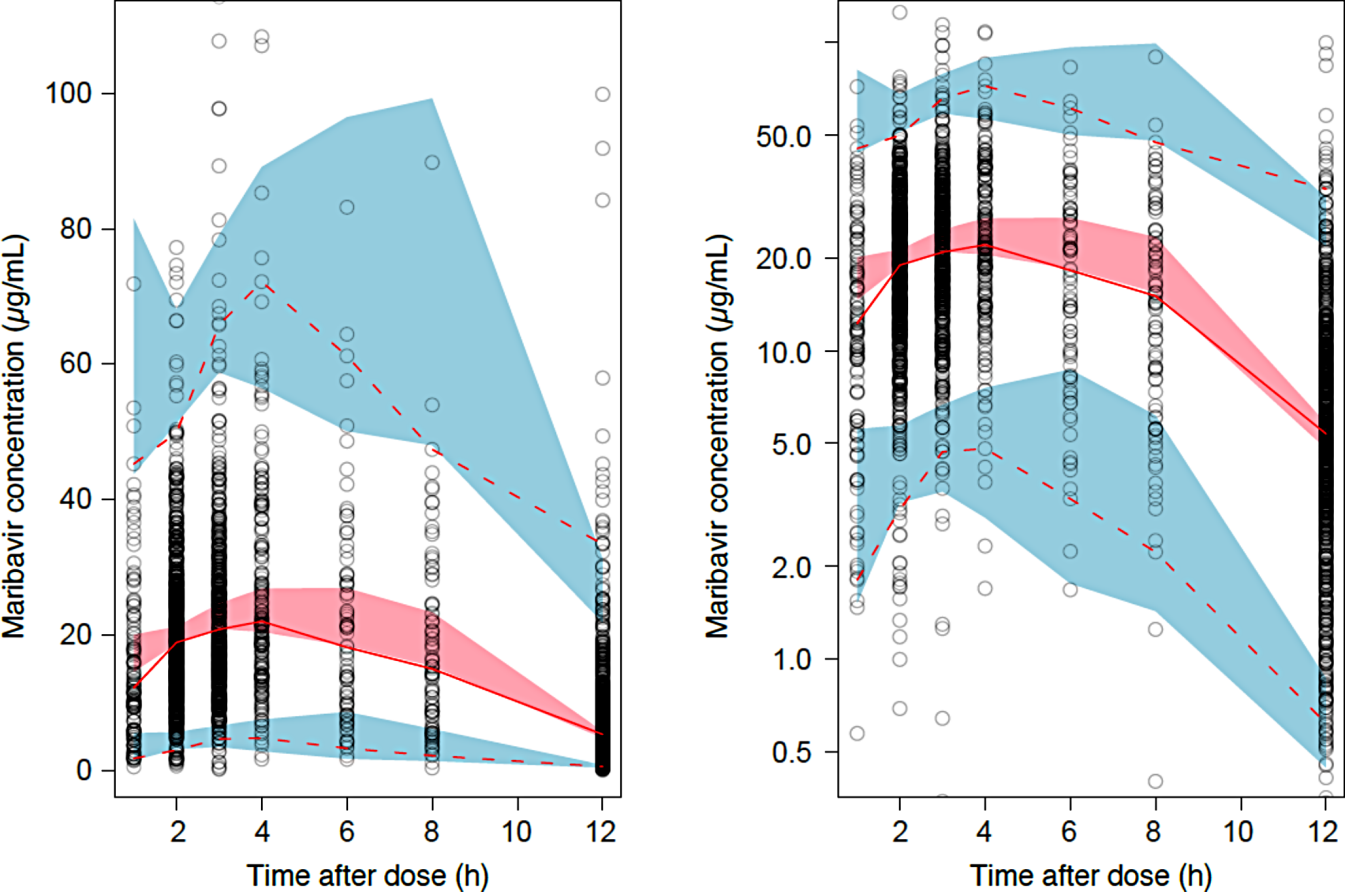
Prediction-corrected visual predictive check for the final population pharmacokinetic model for transplant recipients with cytomegalovirus infection. Left: Linear Y axis scale; Right: Logarithmic Y axis scale. Linear X Scale. Open circle: observed concentrations; solid line: median of observed concentrations; dashed lines: 2.5th and 97.5th percentile of observed concentrations. Red-shaded region: 95% prediction interval for median of predicted concentrations; blue-shaded regions: 95% prediction intervals for the 2.5th and 97.5th percentiles of predicted concentrations. h, hour

Figure 1 shows the results of the CI-pcVPC for transplant recipients with CMV infection. CI-pcVPC showed overall good agreement for the median and 2.5th and 97.5th percentiles of concentrations between observations and predictions. A slight under-prediction was noted in the CI-pcVPC plots for impaired renal function, but this under-prediction was also noted in the diagnostic plots for the healthy volunteers in the renal impairment study and thus is thought to be study-specific rather than due to renal impairment.

Approximately 92% of the observed concentrations fell within the 90% prediction intervals, indicating that the model adequately described the observed data.

#### Estimates of maribavir PK parameters at steady state

The final model for maribavir was used to compute individual estimates of AUC_0-τ_, C_max.ss_, and C_trough.ss_ for all individuals included in the PopPK analysis receiving maribavir 400 mg BID in the absence of strong CYP3A4 inducers or inhibitors. GM AUC_0-τ_ and C_max.ss_ were 27% and 5% higher, respectively, in transplant patients with CMV infection than in healthy volunteers.

**Fig. 2:**
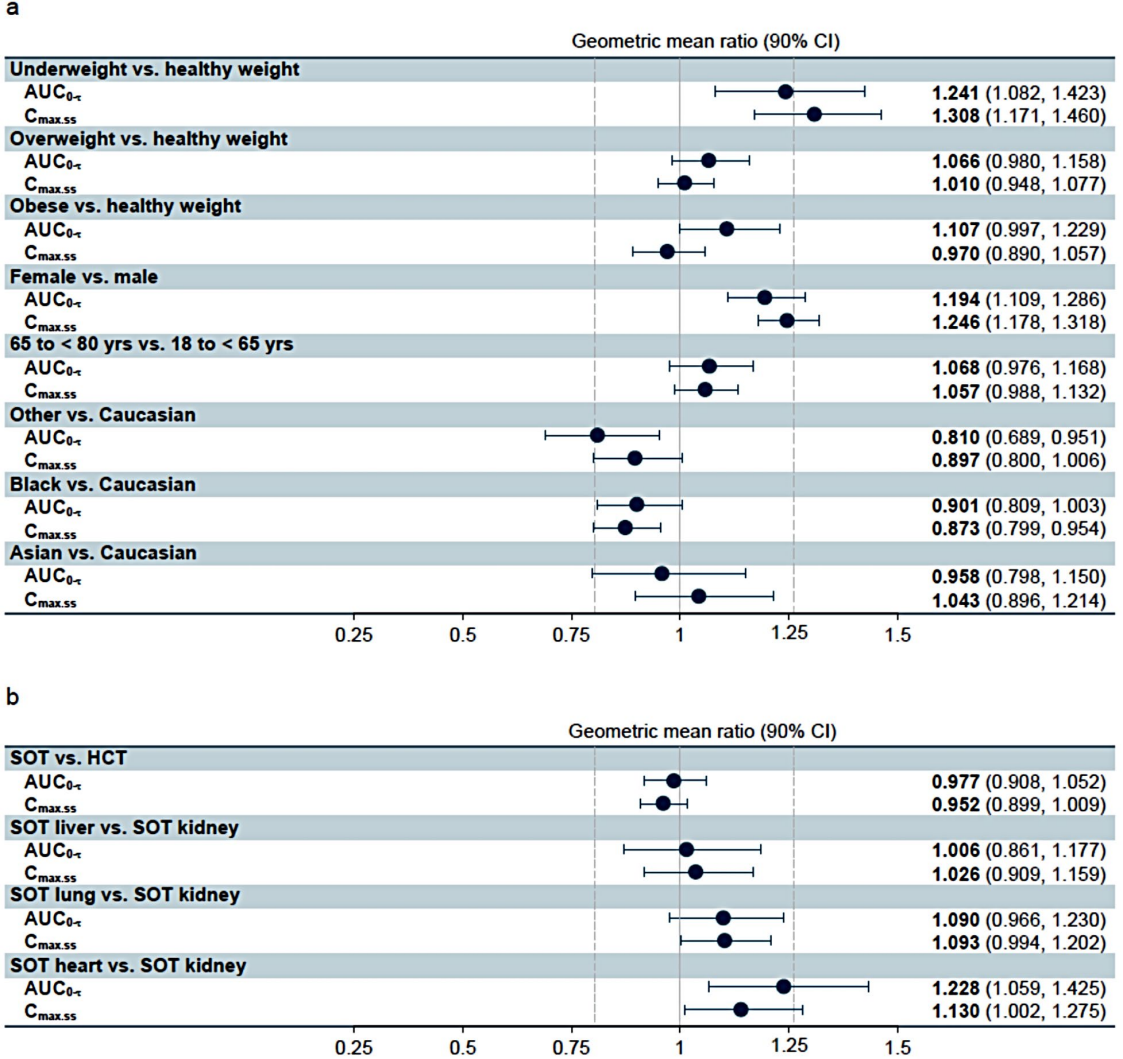
Effect of various intrinsic or extrinsic factors on the steady-state AUC_0−τ_ and C_max.ss_ of maribavir with corresponding geometric mean ratios and 90% confidence intervals in transplant patients with CMV for (**a**) weight, sex, age, and race, and (**b**) transplant type. Underweight = BMI < 18.5 kg/m^2^, healthy weight = BMI 18.5 to < 25 kg/m^2^, overweight = BMI 25 to < 30 kg/m^2^, obese = BMI ≥ 30 kg/m^2^. AUC_0–τ_, area under the concentration–time curve from time 0 to the end of the dosing interval; BMI, body mass index; C_max_, maximum plasma concentration; HCT, hematopoietic cell transplant; SOT, solid organ transplant. Figure a was published in *Transplantation and Cellular Therapy*, 28 3S. Ivy Song, Grace Chen, Siobhan Hayes, Colm Farrell, Claudia Jomphe, Nathalie H Gosselin. Population pharmacokinetics and exposure–response relationships of maribavir in transplant recipients with cytomegalovirus infection. S368-S369, Copyright American Society for Transplantation and Cellular Therapy. Published by Elsevier Inc. (2022). Figure b reproduced from Kefeng Sun, Martha Fournier, Aimee K. Sundberg and Ivy H. Song. Maribavir: mechanism of action, clinical, and translational science. *Clin Transl Sci.* 2024;17(1):10.1111/cts.13696

Maribavir PK parameters for transplant recipients with CMV infection receiving maribavir 400 mg BID are shown in Table 3 and PK parameters for the overall population and for healthy volunteers are shown in Supplementary Table S1.1 of the Supplementary Material 1. The GM ratios and 90% CIs are presented in Fig. 2 for comparisons of AUC_0-τ_ and C_max.ss_ in transplant patients with CMV infection by various patient characteristics. Underweight patients (BMI < 18.5 kg/m^2^) had higher exposure than normal-weight patients (BMI 18.5 to < 25 kg/m^2^), with GM AUC_0-τ_ and C_max.ss_ 24% and 31% higher, respectively. Maribavir exposure was similar in patients who were overweight, obese, or normal-weight, and in patients categorized by race, but female patients had higher exposure than male patients, with GM AUC_0τ_ and C_max.ss_ 19% and 25% higher, respectively. AUC_0-τ_ and C_max.ss_ were comparable in patients aged ≥ 65 years, or 65 to < 75 years, compared with younger patients. In patients aged 75 to < 85 years, AUC_0-τ_ and C_max.ss_ were 54% and 38% higher, respectively, compared with patients aged 18 to < 65 years, although the 90% CIs included 1 and were very wide, reflecting the small number of patients in the 75 to < 85 years age group (*n* = 5). Maribavir exposure was similar with or without PPI, with or without histamine H_2_ blockers, between HCT and SOT patients; between patients receiving kidney, lung, liver, or heart transplants; and additionally between patients with no, Child-Pugh class A, or Child-Pugh class B hepatic impairment, and between patients with or without diarrhea, vomiting, or GI GvHD.

**Table 3:** Summary of steady-state maribavir pharmacokinetic parameters (400 mg BID) in transplant patients with CMV infection

Population	*n*	Geometric mean (%CV)		
		AUC_0– *τ*_ (*µ*g·h/mL)	C_max.ss_ (*µ*g/mL)	C_trough.ss_ (*µ*g/mL)
All transplant patients with CMV infection	485	128 (50.7)	17.2 (39.3)	4.90 (89.7)
Transplant patients with refractory CMV infection (SOLSTICE study only)	253	129 (51.6)	16.9 (39.1)	5.13 (93.0)

AUC_0–τ_, area under the concentration–time curve from time 0 to end of the dosing interval; BID, twice daily; C_max.ss_, maximum maribavir plasma concentration at steady state; CMV, cytomegalovirus; C_trough.ss_, minimum maribavir plasma concentration at steady state; CV, coefficient of variation; h, hour

### Exposure–response analysis

#### Description of observed data and patient characteristics

In total, 231 of the 235 patients with refractory CMV infection from the maribavir arm of the SOLSTICE study had PK exposure parameters and were included in the exposure–response analyses. Patient characteristics are summarized in Table 4, with further detail in Supplementary Table S1.2.

**Table 4:** Summary of patient characteristics in the exposure–response analysis

	Patients (*n* = 231)
Age, years	
18 to < 65	55 (24)
45 to < 65	123 (53)
≥ 65	53 (23)
Mean (SD)	53.7 (13.4)
Median (range)	56.0 (19.0 − 79.0)
Sex	
Male	144 (62)
Female	87 (38)
Race	
White	175 (76)
Black or African American	29 (13)
Asian	9 (4)
Other	16 (7)
Missing/not reported	2 (1)
Weight (kg)	
Mean (SD)	75.7 (17.9)
Median (range)	74.1 (36.1 − 124)
Transplant type	
Solid organ transplant	140 (61)
Hematopoietic cell transplant	91 (39)
CMV symptom status	
Asymptomatic	199 (86)
Symptomatic CMV	14 (6)
Tissue invasive disease	18 (8)
Baseline CMV DNA levels	
Low	151 (65)
Intermediate	66 (29)
High	14 (6)
Treatment-emergent CMV maribavir-resistant mutation	
No	171 (74)
Yes	60 (26)
During maribavir treatment	47 (20)^a^
After maribavir treatment	13 (6)^a^

Data are n (%) unless otherwise specified

CMV, cytomegalovirus

^a^Expressed as a percentage of 60 patients with treatment-emergent CMV mutation conferring resistance to maribavir

#### Exposure–response analysis of efficacy

**Fig. 3:**
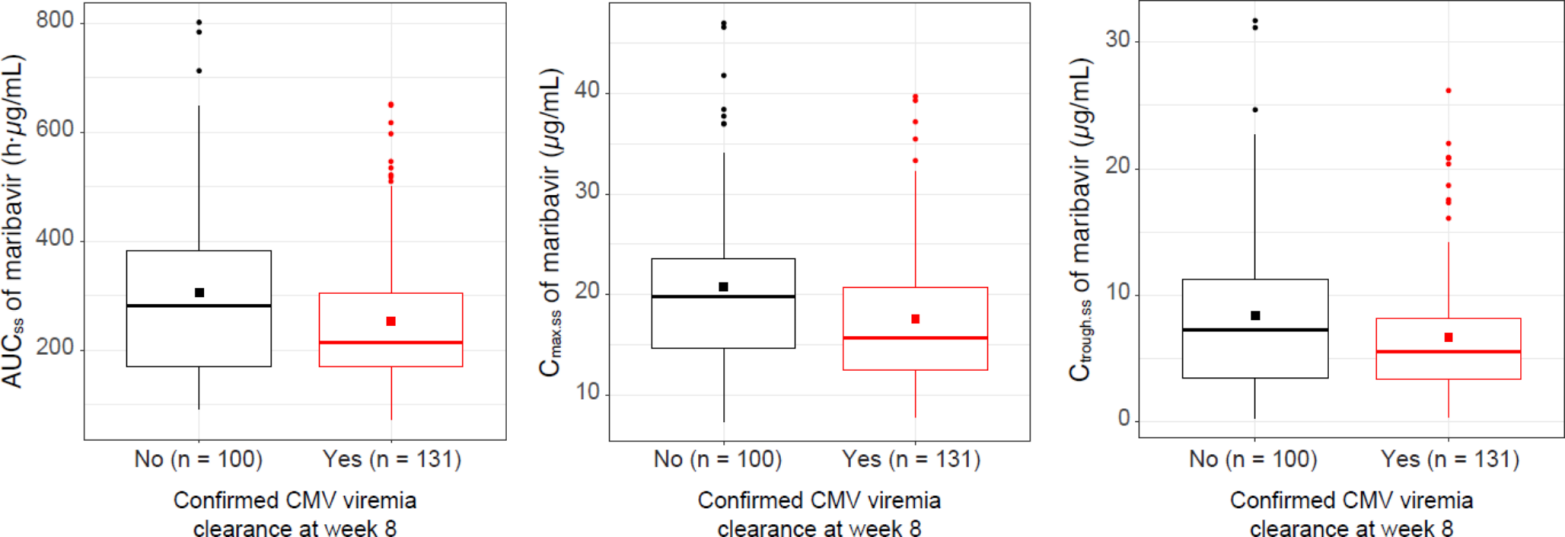
Maribavir exposure in the exposure–efficacy population by primary endpoint response: confirmed CMV clearance at the end of week 8. The lower and upper hinges correspond to the 25th and 75th percentiles, respectively. The upper and lower whiskers extend from the hinge to the largest or smallest value, respectively, no further than 1.5 * IQR from the hinge. Square symbols represent the arithmetic mean, circles represent outliers (i.e. data beyond the end of the whiskers). AUC_ss_, area under the plasma concentration–time curve at steady state on the last day of exposure; C_max.ss_, maximum concentration of maribavir at steady state on the last day of exposure; CMV, cytomegalovirus; C_trough.ss_, minimum concentration of maribavir at steady state on the last day of exposure; IQR, interquartile range

Of the 231 patients included in the analysis, 131 (57%) met the primary endpoint of confirmed CMV viremia clearance at week 8, and 44 (19%) met the key secondary endpoint of confirmed CMV viremia clearance and symptom control at week 8, followed by maintenance through week 16. Box plots of PK parameters for maribavir in responders and non-responders for the primary endpoint are shown in Fig. 3. A total of 60 patients had TE maribavir-resistant CMV mutations (47 were detected on treatment and 13 post treatment).

#### Confirmed clearance of CMV DNA at study week 8

**Fig. 4:**
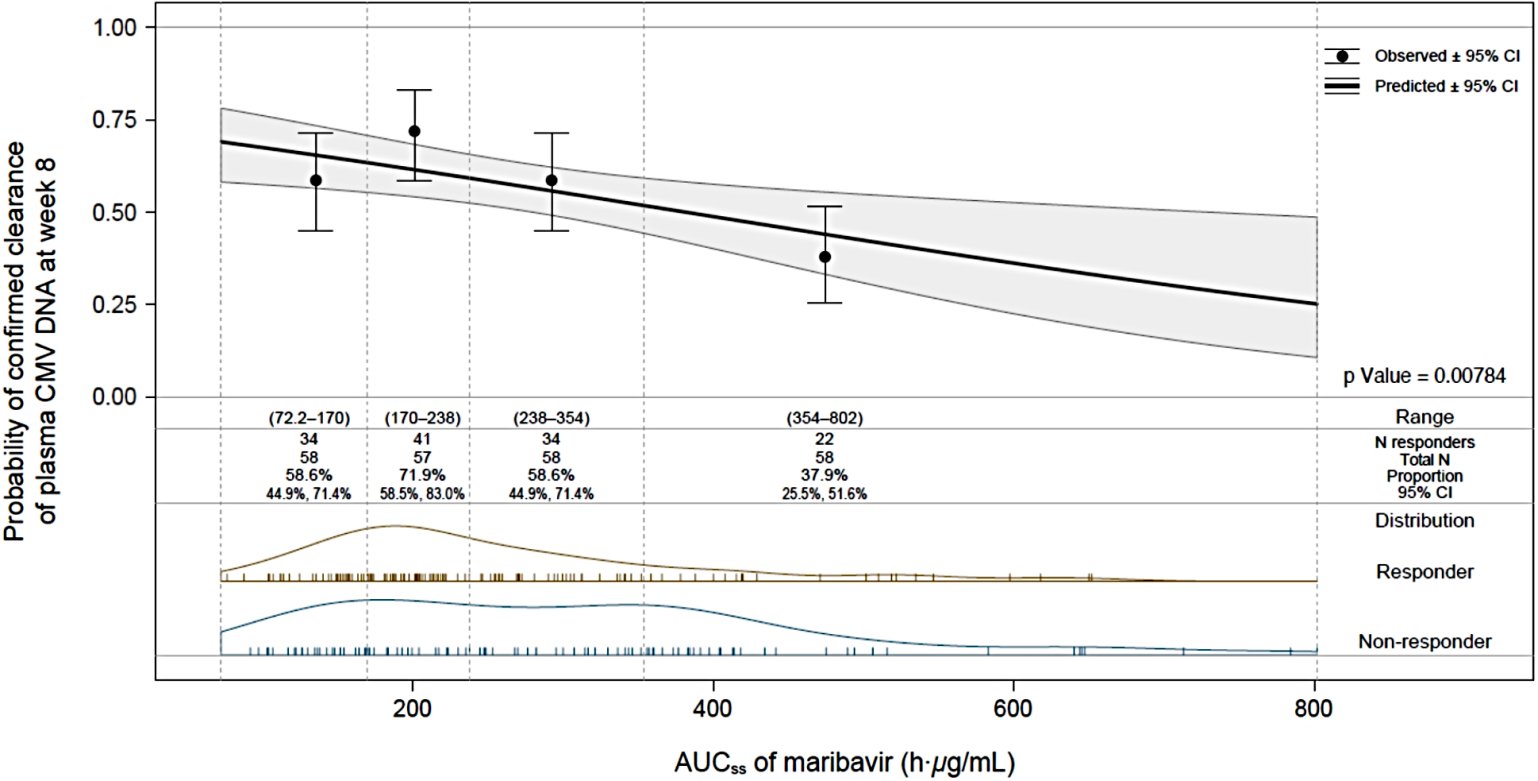
Probability of achieving primary endpoint: confirmed CMV clearance of plasma CMV DNA at week 8, as a function of area under the plasma concentration–time curve at steady state on the last day of exposure. AUC_ss_, area under the plasma concentration–time curve at steady state on the last day of exposure; CI, confidence interval; CMV, cytomegalovirus; N, number of patients; OR, odds ratio

Various exposure–response models were developed to assess the association between maribavir exposure and the probability of confirmed clearance of CMV DNA at study week 8. Although the C_max.ss_ exposure metric was associated with a slightly better statistical goodness-of-fit than the AUC_ss_ metric (AIC = 310.1 and AIC = 312.6, respectively), AUC_ss_ was selected in the model to quantify the impact of maribavir exposure on response and to perform the covariate analysis. An apparent negative relationship between maribavir exposure (AUC_ss_) and probability of CMV viremia clearance at week 8 was observed (*p* = 0.008) (Fig. 4). This relationship was mainly driven by the smaller number of responders in the 4th quartile; the observed proportions of responders increased from the 1st quartile (59%) to the 2nd quartile (72%) and subsequently decreased in the 3rd and 4th quartiles (59% and 38%, respectively). The majority of patients with TE maribavir-resistant CMV mutations were non-responders (81%), and 65% presented in the 3rd and 4th quartiles. In an exploratory analysis performed by excluding 60 patients with TE CMV mutation conferring resistance to maribavir, no statistical exposure–efficacy relationship was observed between maribavir exposure and probability of CMV viremia clearance at week 8 (*n* = 171; *p* = 0.0709).

The covariate analysis identified TE CMV maribavir-resistant mutation during maribavir treatment and CD8 + CD69 + cell count at baseline as other predictive factors for treatment response, in addition to maribavir exposure. Inclusion of CMV resistance at baseline resulted in a lower AIC, but it was not significant as a covariate and so was not retained in the model. No other risk factors were identified.

The final logistic regression model for the exposure–response analysis of the probability of confirmed CMV clearance of plasma CMV DNA at week 8 is presented in Table 5. The intercept was statistically significant, with an estimated exponent of 1.58 on the logit scale (*p* < 0.001). A statistically significant negative exposure–response relationship was observed between maribavir AUC_ss_ and the probability of confirmed CMV clearance of plasma CMV DNA at week 8 with an estimated exponent of − 0.131 on the logit scale 50 *µ*g·h/mL increment (*p* = 0.0233), which corresponds to an OR of 0.877 (95% CI: 0.780, 0.982). The presence of TE CMV maribavir-resistant mutations had a clinically significant impact on response, with transplant recipients who developed TE CMV maribavir-resistant mutation having a 1.3% probability of being responders compared with 74.2% of transplant recipients who did not develop a TE CMV maribavir-resistant mutation, assuming a CD8 + CD69 + cell count at baseline < 0.5% and AUC_ss_ of 200 *µ*g∙h/mL. The impact of a CD8 + CD69 + cell count ≥ 0.5% and < 2% was not statistically significant: transplant recipients with CD8 + CD69 + cell count of ≥ 2% had a 90.0% probability of being responders compared with 74.2% for those with CD8 + CD69 + cell count at baseline < 0.5%, assuming no development of TE CMV maribavir-resistant mutation and AUC_ss_ of 200 *µ*g∙h/mL.

**Table 5:** Logistic regression parameters for primary endpoint: confirmed CMV clearance of plasma CMV DNA at week 8

Parameters	Estimate (SE)	OR (95% CI)	*p* Value
Intercept	1.58 (0.413)	–	< 0.001
AUC_ss_ of maribavir – increment of 50 *µ*g·h/mL	− 0.131 (0.0588)	0.877 (0.780, 0.982)	0.0233
Treatment-emergent maribavir resistance on maribavir treatment	− 5.39 (1.42)	0.00457 (3.59 × 10^–5^, 0.0333)	< 0.001
Baseline CD8 + CD69 + cell count			
≥ 0.5% to < 2%	− 0.304 (0.492)	0.739 (0.289, 1.98)	0.535
≥ 2%	1.19 (0.630)	3.29 (1.07, 13.4)	0.0371
Not reported	− 0.314 (0.415)	0.730 (0.328, 1.66)	0.447

Reference subject did not develop treatment-emergent maribavir resistance during treatment, had CD8 + CD69 + cell count of < 0.5% at baseline, and did not present CMV resistance at baseline

AUC_ss_, area under the plasma concentration–time curve at steady state on the last day of exposure; CI, confidence interval; CMV, cytomegalovirus; h, hour; OR, odds ratio; SE, standard error

#### Confirmed CMV viremia clearance and CMV infection symptom control at week 8 followed by maintenance through week 16

Of all the models tested for the key secondary endpoint, confirmed CMV viremia clearance and CMV infection symptom control at week 8 followed by maintenance through week 16, maribavir AUC_ss_ was associated with the best statistical goodness-of-fit (AIC = 214.6) in the logistic regression model. Box plots for PK parameters for maribavir in responders and non-responders for the key secondary endpoint are shown in Supplementary Fig. S1.1. An apparent negative relationship between maribavir exposure (AUC_ss_) and probability of response for the key secondary endpoint was observed (*p* = 0.001), with small proportions of responders (31%, 25%, 16%, and 5% for the 1st, 2nd, 3rd, and 4th quartiles of exposure, respectively) (Supplementary Fig. S1.2). It should be noted, however, that there were small numbers of responders in the 3rd and 4th quartiles (*n* = 9 and *n* = 3, respectively).

Significant predictive factors identified in the covariate analysis were TE CMV maribavir-resistant mutation, CD8 + CD69 + cell count at baseline, CMV DNA level at baseline, and prior use of CMV prophylaxis. The final logistic regression model is presented in Supplementary Table S1.3. Transplant recipients who developed TE CMV maribavir-resistant mutation had a 2.5% probability of being a responder versus 40.3% in those who did not develop TE CMV maribavir-resistant mutation, assuming CD8 + CD69 + cell count at baseline < 0.5%, low CMV DNA levels, no prior use of CMV prophylaxis, and AUC_ss_ of 200 *µ*g∙h/mL. Patients with baseline CD8 + CD69 + cell count ≥ 2% had a higher probability of being a responder than those with a CD8 + CD69 + cell count of < 0.5% (77.9% vs. 40.3%, respectively), assuming no development of TE CMV maribavir-resistant mutation, low CMV DNA levels, no prior use of CMV prophylaxis, and AUC_ss_ of 200 *µ*g∙h/mL. Transplant recipients with intermediate/high CMV DNA levels had a 19.7% probability of being a responder, versus 40.3% in those with low CMV DNA levels, assuming CD8 + CD69 + cell count < 0.5%, no development of TE CMV maribavir-resistant mutation, no prior use of CMV prophylaxis, and AUC_ss_ of 200 *µ*g∙h/mL. Transplant recipients with prior use of CMV prophylaxis had a 23.0% probability of being responder versus 40.3% in those without any use of CMV prophylaxis therapy, assuming no development of TE CMV maribavir-resistant mutation, CD8 + CD69 + cell count < 0.5%, low CMV DNA level, and AUC_ss_ of 200 *µ*g·h/mL.

#### Relationship between maribavir exposure and development of treatment-emergent CMV mutations conferring resistance to maribavir

TE maribavir-resistant CMV mutations were identified in 47 patients during the 8-week treatment period, all of which were reported in non-responders (i.e., those not achieving viremia clearance at week 8; *n* = 47/100 [47%]). TE CMV maribavir-resistant mutations were identified in 13 patients after end of treatment.

**Fig. 5:**
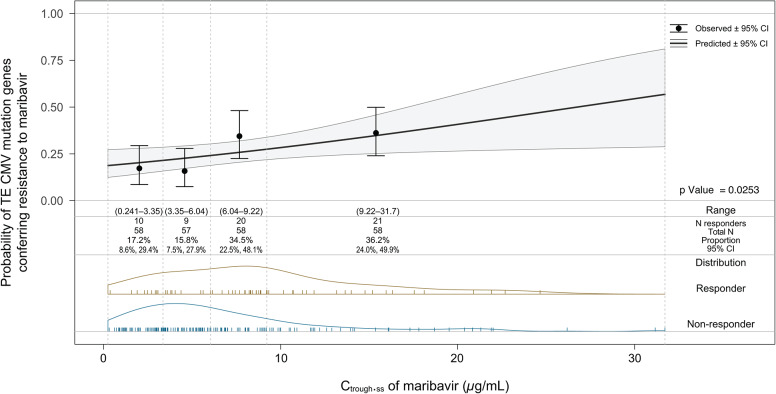
Probability of developing treatment-emergent CMV mutations conferring resistance to maribavir as a function of minimum plasma concentration at steady state on the last day of exposure. CI, confidence interval; CMV, cytomegalovirus; C_trough.ss_, minimum plasma concentration at steady state on the last day of exposure; OR, odds ratio; TE, treatment-emergent

Of all models tested, maribavir C_trough.ss_ was associated with the best statistical goodness-of-fit in the logistic regression model (AIC = 263.6817). A positive relationship was observed between maribavir exposure (C_trough.ss_) and probability of developing TE CMV maribavir-resistant mutations (*p* = 0.0253) (Fig. 5). Significant risk factors identified in the covariate analysis were CMV DNA level at baseline, CMV resistance at baseline, CD4 + CD69 + cell count at baseline, age, and transplant type. The final logistic regression model is presented in Table 6. HCT transplant recipients with intermediate and high CMV DNA levels had a 70.5% and 84.5% probability of developing TE maribavir-resistant mutations, respectively, versus 54.5% in transplant recipients with low CMV DNA levels of the same age, assuming the absence of CMV resistance at baseline, a CD4 + CD69 + cell count < 0.5%, and C_trough.ss_ of 5 *µ*g∙h/mL. HCT transplant recipients with CMV resistance at baseline had a 28.9% probability of developing a TE maribavir-resistant mutation, versus 54.4% in transplant recipients of the same age who did not have CMV resistance at baseline, assuming low CMV DNA levels, CD4 + CD69 + cell count at baseline < 0.5%, low CMV DNA levels, and C_trough.ss_ of 5 *µ*g∙h/mL. HCT transplant recipients with CD4 + CD69 + cell count of ≥ 0.5% and < 2% had a 20.7% probability of developing TE maribavir-resistant mutations, versus 54.5% in transplant recipients of the same age with CD4 + CD69 + cell count at baseline < 0.5%, assuming the absence of CMV resistance at baseline, low CMV DNA levels, and C_trough.ss_ of 5 *µ*g∙h/mL. There was a decrease in the probability of a TE maribavir-resistant mutation of 0.8% by year of age. SOT recipients had a 75.9% probability of developing TE maribavir-resistant mutations, versus 54.4% in HCT recipients with low CMV DNA levels, assuming the absence of CMV resistance at baseline, CD4 + CD69 + cell count < 0.5% and C_trough.ss_ of 5 *µ*g∙h/mL.

To rule out the possibility that TE CMV mutations had caused the higher maribavir exposure, a complementary analysis was conducted by examining observed predose (trough) maribavir concentrations (C_min_) in SOLSTICE by PK visits (weeks 1, 4, and 8) and by occurrence of on-treatment TE CMV mutation status. Median observed C_min_ values in transplant recipients who developed TE CMV mutation conferring resistance to maribavir while receiving treatment were consistently higher than those observed in transplant recipients without TE CMV mutation across weeks 1, 4, and 8.

**Table 6:** Logistic regression parameters for development of treatment-emergent mutations conferring resistance to maribavir

Parameters	Estimate (SE)	OR (95% CI)	*p* Value
Intercept	− 0.242 (0.711)	–	0.733
C_trough.ss_ of maribavir – increment of 2.5 *µ*g/mL	0.210 (0.0762)	1.23 (1.06, 1.43)	0.006
Baseline CMV DNA levels			
Intermediate	0.694 (0.364)	2.00 (0.982, 4.08)	0.056
High	1.52 (0.632)	4.57 (1.32, 15.8)	0.016
Baseline CMV resistant	− 1.08 (0.388)	0.341 (0.159, 0.729)	0.006
Baseline CD4 + CD69 + cell count			
≥ 0.5% to < 2%	− 1.52 (0.594)	0.219 (0.0683, 0.700)	0.010
≥ 2%	− 0.0534 (0.768)	0.948 (0.210, 4.27)	0.945
Not reported	− 0.185 (0.428)	0.831 (0.359, 1.92)	0.666
Age	− 0.0328 (0.0129)	0.968 (0.944, 0.993)	0.011
Solid organ transplant	0.970 (0.413)	2.64 (1.17, 5.92)	0.019

Reference subject had low CMV DNA level at baseline, was not CMV resistant at baseline, had a CD4 + CD69 + cell count < 0.5%, and received a hematopoietic stem cell transplant

CI, confidence interval; CMV, cytomegalovirus; C_trough.ss_, minimum maribavir plasma concentration at steady state on the last day of exposure; OR, odds ratio; SE, standard error

#### Relationship between maribavir exposure and probability of TEAEs

Among all AEs evaluated, a statistically significant association with maribavir exposure was identified for taste disturbance, fatigue, and TE SAE.

A statistically significant negative relationship (*p* < 0.05) was observed between probability of taste disturbance and C_avg_ of maribavir (Supplementary Fig. S1.3). Logistic regression identified transplant type and enrollment region as statistically significant risk factors for taste disturbance.

Statistically significant positive relationships were observed between the probability of fatigue and all maribavir exposure PK metrics with the strongest relationship observed with C_max, ss_ (Supplementary Fig. S1.4). Logistic regression identified enrolling region as a statistically significant risk factor for fatigue.

Statistically significant positive relationships were also observed between probability of TE SAE and maribavir AUC_day_, AUC_ss_, C_max.ss_, and C_avg_. Of all models tested, the AUC_ss_ of maribavir was associated with the best statistical goodness-of-fit. Logistic regression identified CMV resistance at baseline and CMV DNA level at baseline as statistically significant risk factors for TE SAE (Supplementary Fig. S1.5).

## Discussion

These PopPK and exposure–response analyses were conducted to provide additional evidence in support of the recommended 400 mg BID daily dose for maribavir. Overall, the findings support the recommended dose in transplant recipients with refractory CMV infection, including in special populations.

In the PopPK analysis, maribavir PK was well described by a two-compartment disposition model with first-order elimination, first-order absorption, and an absorption lag-time. The first-order absorption rate constant decreased with increasing dose (400, 800, and 1200 mg doses). As expected, administration of maribavir with strong CYP3A4 inhibitors had a significant impact on maribavir PK, with clearance reduced by 30%. This is consistent with the previous finding that ketoconazole increased maribavir AUC_0-∞_ and C_max_ by 53% and 10%, respectively [22]. Similarly, administration of maribavir with strong CYP3A inducers had a significant impact on maribavir PK, with a 2.24-fold increase in clearance. Again, this is consistent with previous findings of a 60% reduction in AUC, 30% reduction in half-life, and 2.5-fold increase in clearance when maribavir was administered with rifampin [23].

Clearance was 24% lower for transplant patients with CMV infection than for all other individuals, whereas AUC_0–τ_ and C_max.ss_ were 27% and 5% higher in transplant patients with CMV infection than in healthy volunteers. These differences are potentially due to the decreased liver/kidney function in the transplant patient populations and use of concurrent medications.

The clinical relevance of the impact from various intrinsic or extrinsic factors was defined by the “no-effect” boundaries. The lower “no-effect” boundary was set at the maribavir AUC and C_trough_ levels at 80% of the corresponding values following maribavir 400 mg BID in transplant patients to ensure antiviral activity of the drug. The upper “no-effect” boundary was set at the AUC and C_max_ at 1200 mg BID in transplant patients, as this dose was associated with a similar level of TEAEs as lower doses in phase 2 studies [17, 18]. Individual exposure estimates indicated that steady-state AUC_0–τ_ and C_max.ss_ were higher in individuals with BMI < 18.5 kg/m^2^ than in those with BMI 18.5 to < 25 kg/m^2^, but similar in those with BMI 18.5 to < 25 kg/m^2^, 25 to < 30 kg/m^2^, and ≥ 30 kg/m^2^ (Fig. 2). These differences are not considered clinically relevant given the wide therapeutic window of maribavir (i.e., similar anti-CMV efficacy and similar rate of the most common AE, dysgeusia, at doses of 400–1200 mg BID) [17, 18]. Furthermore, there was no evidence that age, age category, sex, race, or ethnicity affected maribavir PK (Fig. 2). In a previous publication, systemic maribavir exposure was found to be higher in healthy Japanese individuals compared with White individuals [31], but the authors did not consider the difference to be clinically relevant. Other variables that were not identified as significant covariates for maribavir PK in the present analysis were diarrhea, vomiting, disease characteristics, H_2_ blockers, and PPI. In addition, the PK of maribavir was unaffected in patients with mild or moderate hepatic impairment and no dose reduction is necessary [10, 11, 32]. Consistent with the minimal renal clearance of maribavir, no dose adjustment is required for patients with mild, moderate, or severe renal impairment [10, 11, 33].

For antiviral therapy, durability of response and risk of development of resistance leading to loss of response are of special interest. For these reasons, the therapeutic dose is usually targeted at maximal tolerable dose(s). In the two phase 2 studies in transplant recipients with refractory CMV infection, the incidence of dysgeusia (the most frequently reported TEAE) was similar and there was no evidence of dose-related myelosuppression at doses up to 1200 mg BID [17, 18]. The pivotal phase 3 study, SOLSTICE, evaluated a lower dose of 400 mg BID [9]. Although maribavir demonstrated superior efficacy and an improved safety profile over other CMV therapies in the SOLSTICE study [9], there was a natural question as to whether a dose higher than 400 mg BID would provide better durability of response and reduce development of resistance. The SOLSTICE study incorporated an extensive evaluation of CMV resistance [15], providing a more complete genotypic dataset than the two phase 2 studies [16]. The current exposure–response analysis for viremia clearance and development of CMV mutations conferring resistance to maribavir in SOLSTICE can help answer the question of the potential (or lack of) benefit of higher doses.

Overall, data from SOLSTICE indicated that higher maribavir drug exposure was not associated with higher antiviral response rate, nor with lower rate of maribavir-resistant mutations, suggesting doses higher than 400 mg BID would not provide additional efficacy benefits. The exposure–response analysis identified a statistically significant negative association between maribavir exposure and CMV viremia clearance at week 8 (Fig. 4), which was unexpected. Upon further evaluation and considering TE resistance, it became apparent that these negative associations were mostly driven by the higher number of patients with TE resistance in the two higher quartiles of maribavir exposure. When examining data only in patients without TE resistance to maribavir, the association between maribavir exposure and antiviral response became statistically insignificant, suggesting a flat exposure–response relationship and that a dose higher than 400 mg BID does not indicate a higher rate of viremia clearance. It is not uncommon to observe a flat exposure–response relationship with antiviral drugs in pivotal clinical trials. For example, no significant exposure dependencies were found for clinically significant CMV infection (CS-CMVi) through Week 24 or Week 14 among letermovir-treated participants. Furthermore, the covariates evaluated had no impact on exposure-CS-CMVi relationships, and letermovir exposure did not affect time of onset for CS-CMVi [28].

The exposure–response analysis of TE resistance revealed a positive association between maribavir exposure (C_trough.ss_) and TE mutations conferring resistance to maribavir (Fig. 5), suggesting higher maribavir drug concentrations may lead to increased probability of resistance development. The reason for this positive association is unclear, although it could be hypothesized that the inability of anti-CMV drugs to kill the virus may ultimately select resistant strains [4]. The higher level in C_trough_ in transplant patients with TE resistance compared with patients without TE resistance was apparent in week 1 and consistent throughout the 8-week treatment period, suggesting the higher exposure in the patients with TE resistance did not result from emerging UL97 mutation. A few other risk factors were identified, including baseline CMV DNA levels, CMV resistance at baseline, CMV-specific CD4 + CD69 + cell count at baseline, age, and transplant type. Taking these into consideration, maribavir doses higher than 400 mg BID are unlikely to reduce the risk of development of resistance to maribavir in the transplant patients with refractory and resistant CMV infections/diseases. This was also observed in the phase 2 maribavir study involving doses of 400–1200 mg BID [16].

A number of limitations should be taken into account when considering the results of the present analyses. While body weight was not found to be a significant predictor of maribavir PK, it was retained in the model because it was used to predict the concentration–time profiles and support dose selection in a pediatric population for an ongoing phase 3 study in pediatric transplant patients with CMV infections. This PopPK model will be further validated and refined upon availability of pediatric data. However, based on the CI-pcVPC and goodness-of-fit evaluation, the current PopPK model is adequate to characterize the maribavir PK in adults, and exposure data generated using the post hoc PK parameters from the PopPK model are suitable for the exposure–response analysis. In addition, although the PopPK analysis showed a lack of difference of PK parameter values between patients with and without GI GvHD, the overall instances of GI GvHD were low (*n* = 12).

It should be noted that exposure levels at the last day were retained as a predictor of maribavir efficacy but may not represent the overall exposure during the trial, including potential dosing adjustment or holiday periods. Moreover, collinearity or interactions among model parameters of logistic regression were not evaluated during the covariate selection process. Another potential limitation of our analysis is the low number of patients with hepatic impairment included (1% of the overall PopPK analysis population), which may affect the applicability of results in these patients.

## Conclusions

In conclusion, results from these analyses suggest that dose adjustment of maribavir is not required for the treatment of CMV infection in adult transplant recipients, regardless of age (> 65 years vs. ≤ 65 years), body weight (underweight, normal weight, overweight, or obese), sex, race, transplant type, baseline plasma CMV DNA, presence of CMV mutations, ongoing diarrhea, or GI GvHD. Higher maribavir drug exposure may not result in higher rates of CMV viremia clearance nor a reduced rate of development of maribavir resistance, suggesting doses higher than 400 mg BID will unlikely provide additional clinical benefit. Overall, the findings of these PopPK and exposure–response analyses provide further support for the recommended maribavir dose of 400 mg BID.

## Electronic supplementary material

Below is the link to the electronic supplementary material.

Supplementary Material 1

## Data Availability

The datasets, including the redacted study protocol, redacted statistical analysis plan, and individual participants data supporting the results reported in this article, will be made available, within three months from initial request, to researchers who provide a methodologically sound proposal. The data will be provided after its de-identification, in compliance with applicable privacy laws, data protection and requirements for consent and anonymization.

## Abbreviations

α: Baseline Ln (odds) of the event
AE: Adverse event
AIC: Akaike information criterion
AUC: Area under the concentration–time curve
AUC_0-∞_: AUC from time 0 to infinity
AUC_0–τ_: AUC at steady state from time 0 to end of the dosing interval
AUC_day_: AUC from 0 to 24 h on the day of an AE
AUC_ss_: AUC from 0 to 24 h at steady state on the last day of exposure
β_1_: Slope linking the exposure parameter (X_1_)
BID: Twice daily
BLQ: Below the lower limit of quantification
BMI: Body mass index
C_avg_: Average concentration on each study day
CI: Confidence interval
CI-pcVPC: Confidence interval prediction-corrected visual predictive check
CL/F: Total clearance
C_max_: Maximum plasma concentration
C_max.day _: C_max_ on the day of an AE
C_max.ss_: Maximum concentration at steady state
C_min_: Predose plasma concentrations
CMV: Cytomegalovirus
C_trough.ss_: Minimum plasma concentration at steady state
CV: Coefficient of variation
CV%: Percentage CV
CYP: Cytochrome P450
D: Donor
GI: Gastrointestinal
GM: Geometric mean
GvHD: Graft-versus-host disease
HCT: Hematopoietic cell transplant
IAT: Investigator-assigned therapy
IIV: Inter-individual variability
Ka: First-order absorption rate
NONMEM: Non-linear mixed effects modeling
OR: Odds ratio
P_i_: Probability of the event in the ith patient
PK: Pharmacokinetics
PopPK: Population pharmacokinetic
PPI: Proton pump inhibitors
Q/F: Inter-compartment clearance between central and peripheral compartments
R: Recipient
RSE: Relative standard error
SAEs: Serious AEs
SD: Standard deviation
σ2_prop_: Proportional residual error
SOT: Solid organ transplant
TE: Treatment-emergent
TEAEs: Treatment-emergent adverse events
Vc/F: Apparent volume of central compartment
Vp/F: Apparent volume of peripheral compartment
