# Supplementary material for: Population pharmacokinetics and exposure–response relationships of maribavir in transplant recipients with cytomegalovirus infection
Source: J Pharmacokinet Pharmacodyn. 2024 Sep 27;51(6):887–904. doi: 10.1007/s10928-024-09939-2 (PMC11579209; doi:10.1007/s10928-024-09939-2)
Supplement: Supplementary file 1 — Supplementary Material 1 [file 10928_2024_9939_MOESM1_ESM.docx]

Online Supplementary Material 1

Population pharmacokinetics and exposure–response relationships of maribavir in transplant recipients with cytomegalovirus infection

Ivy H. Song • Grace Chen • Siobhan Hayes • Colm Farrell • Claudia Jomphe • Nathalie H. Gosselin • Kefeng Sun

**Journal**:

*Journal of Pharmacokinetics and Pharmacodynamics*

**Correspondence:**

**Name:** Ivy H. Song

**Affiliation:** Takeda Development Center Americas, Inc., Cambridge, MA, USA

**E-mail:** [ivy.song@takeda.com](mailto:ivy.song@takeda.com)

**Supplementary Methods S1**

**NONMEM control stream**

;Model Desc: Run 156 final 303 data

;Project Name: MaribavirPopPK

;Project ID: MaribavirPopPK

$PROB RUN# 171

$ABBR PROTECT

$INPUT C ID STUDY SITE DAY WK NTIM TIME TAD AMT DROP DROP SS II EVID MDV DV BLQ LLOQ DOSTRT DOSE

FULLPK AGE AGEGT65 SEXN RACEN HTBL WTBL BMIBL TRANSTYP CMVDNAN PRCMVN CMVCATN CMANTIN EQCMVN

ANTICMVN HEPN SUBJID=DROP CYP3AINH CYP3AIND PPI H2B AACID TABL FOOD VOM DIAR CRUSH GIGVHD HS AGEC PERIOD ROWNUM=DROP IMPTIME

$DATA maribavir_202_203_final303_phase1_pk_v1.csv IGNORE=C IGNORE=(BLQ=1)

$SUBROUTINES ADVAN4 TRANS4

$PK

CLWT=LOG(WTBL/70)*THETA(7)

VCWT=LOG(WTBL/70)*THETA(8)

QWT=LOG(WTBL/70)*THETA(9)

VPWT=LOG(WTBL/70)*THETA(10)

HEPN2=0

IF(HEPN.EQ.2) HEPN2=1;Child-Pugh Class B

KADOSE=(DOSE/800)**THETA(16);dose time changing for a few subjects

;HV reference

HSCMV=0

IF(HS.EQ.2) HSCMV=1

MU_1=THETA(1)+CLWT+THETA(14)*SEXN+THETA(17)*HSCMV

CL=EXP(MU_1+ ETA(1))*(THETA(11)**CYP3AINH)*(THETA(12)**CYP3AIND);CYP3AINH and CYP3AIND time-varying

MU_2=THETA(2)+VCWT+THETA(13)*HEPN2+THETA(15)*SEXN

V2=EXP(MU_2+ ETA(2))

MU_3=THETA(3)+QWT

Q=EXP(MU_3+ ETA(3))

MU_4=THETA(4)+VPWT

V3=EXP(MU_4+ ETA(4))

MU_5=THETA(5)

KA=EXP(MU_5+ ETA(5))*KADOSE

MU_6=THETA(6)

ALAG1=EXP(MU_6+ ETA(6))

S2=V2

$ERROR

IPRED = F

Y = IPRED + EPS(1)*IPRED + EPS(2)

IF(STUDY.EQ.202.OR.STUDY.EQ.203.OR.STUDY.EQ.303) Y = IPRED + EPS(3)*IPRED + EPS(2)

$THETA

1.2 ;[ln__CL]

3.0 ;[ln_V2]

0.19 ;[ln_Q]

2.3 ;[ln_V3]

0.35;[ln_Ka]

-1.3 ;[ln_ALAG1]

(0.75 FIX) ;[CL~WT]

(1 FIX) ;[Vc~WT]

(0.75 FIX) ;[Q~WT]

(1 FIX) ;[Vp~WT]

(0, 0.7) ;[CL~CYP3AINH]

(0, 2.3) ;[CL~CYP3AIND]

(0 FIX) ;[Vc~Child-Pugh Class B]

(0 FIX) ;[CL~sex]

(0 FIX) ;[Vc~sex]

(0.01) ;[KA~dose]

(0.01) ;[CL~CMV]

$OMEGA BLOCK(6)

0.1;[P]

0.01;[F]

0.1;[P]

0.01;[F]

0.01;[F]

0.1;[P]

0.01;[F]

0.01;[F]

0.01;[F]

0.1;[P]

0.01;[F]

0.01;[F]

0.01;[F]

0.01;[F]

0.1 ;[P]

0.01;[F]

0.01;[F]

0.01;[F]

0.01;[F]

0.01;[F]

0.04 ;[P]

$SIGMA

0.1 ;[P]

0 FIX ;[A]

0.1 ;[P] studies 202 & 203 & 303

$EST METHOD=IMPMAP INTER EONLY=0 NITER=1000 ISAMPLE=1000 PRINT=1 SIGL=6 NOPRIOR=1 NOHABORT RANMETHOD=3S2 CTYPE=3 NSIG=2 MSFO=171.msf

$COV MATRIX=R PRINT=E UNCONDITIONAL

$TABLE NOPRINT ONEHEADER FILE=171.TAB FORMAT=s1PE16.9

ID STUDY TIME TAD PERIOD WK AMT EVID MDV IPRED CWRES

KA V2 Q V3 CL ALAG1

ETAS(1:LAST) DOSE DOSTRT AGE AGEGT65 SEXN RACEN WTBL BMIBL TRANSTYP CMVDNAN PRCMVN CMVCATN CMANTIN EQCMVN

ANTICMVN HEPN CYP3AINH CYP3AIND PPI H2B AACID TABL FOOD VOM DIAR CRUSH GIGVHD HS AGEC IMPTIME

**Supplementary Table S1.1** Summary of steady-state maribavir pharmacokinetic parameters (400 mg BID) in the overall population and in healthy volunteers

| Population | n | Geometric mean (%CV) | | | |
| --- | --- | --- | --- | --- | --- |
|  |  | AUC_0–_ *_τ_* (*μ*g·hour/mL) | C_max.ss_  (*μ*g/mL) | C_trough.ss_  (*μ*g/mL) | Half-life (hours) |
| All participants | 667 | 121 (48.7) | 17.2 (37.2) | 4.33 (89.7) | 10.9 (27.9) |
| Healthy volunteers | 133 | 101 (37.0) | 16.4 (28.6) | 2.89 (71.7) | 9.04 (28.7) |

AUC_0–τ_, area under the concentration–time curve from time 0 to the end of the dosing interval; BID, twice daily; C_max.ss_, maximum concentration of maribavir at steady state on the last day of exposure; CMV, cytomegalovirus; C_trough.ss_, minimum concentration of maribavir at steady state on the last day of exposure; CV, coefficient of variation.

**Supplementary Table S1.2** Summary of additional patient characteristics in the exposure–response analysis

| Covariate, n (%) | PK set (n = 231) |
| --- | --- |
| Ethnicity |  |
| Hispanic/Latino | 14 (6) |
| Not Hispanic | 194 (84) |
| Unknown/not reported | 23 (10) |
| Antilymphocyte use |  |
| No | 132 (57) |
| Yes | 99 (43) |
| Immune function status (white blood cells) (×10^9^/L) |  |
| < 2.7 | 68 (29) |
| ≥ 2.7 to <7 | 132 (57) |
| ≥ 7 | 18 (8) |
| Not reported | 13 (6) |
| CD4+CD69+ cell count at baseline |  |
| < 0.5%^a^ | 131 (57) |
| ≥ 0.5% to < 2% | 42 (18) |
| ≥ 2% | 12 (5) |
| Not reported | 46 (20) |
| CD8+CD69+ cell count at baseline |  |
| < 0.5%^a^ | 128 (55) |
| ≥ 0.5% to < 2% | 28 (12) |
| ≥ 2% | 29 (13) |
| Not reported | 46 (20) |
| CMV resistant at baseline |  |
| No | 126 (55) |
| Yes | 100 (43) |
| Not reported | 5 (2) |
| CMV serostatus |  |
| D+/R− | 124 (54) |
| D+/R+ | 52 (23) |
| D−/R+ | 41 (18) |
| D−/R− | 12 (5) |
| Missing | 2 (1) |
| Prior CMV prophylaxis |  |
| No | 133 (58) |
| Yes | 98 (42) |

CMV, cytomegalovirus; D, donor; R, recipient

^a^ CD4+CD69+ and CD8+CD69+ cell counts at baseline reported as “too few cells for analysis” were assigned to the <0.5% category

**Supplementary Table S1.3** Logistic regression parameters for key secondary endpoint: confirmed CMV clearance of plasma CMV DNA and CMV infection symptom control at week 8, followed by maintenance through week 16

| Parameters | Estimate (SE) | OR (95% CI) | p Value |
| --- | --- | --- | --- |
| Intercept | 0.814 (0.543) | – | 0.128 |
| AUC_ss_ of maribavir – increment of 50 *µ*g·h/mL | – 0.301 (0.0884) | 0.740 (0.611, 0.870) | <0.001 |
| Treatment-emergent CMV mutation conferring resistance to maribavir | – 3.27 (1.39) | 0.0381 (2.97×10^–4^, 0.284) | <0.001 |
| Baseline CD8+CD69+ cell count |  |  |  |
| ≥0.5% to <2% | – 0.521 (0.624) | 0.594 (0.159, 1.88) | 0.387 |
| ≥2% | 1.65 (0.552) | 5.18 (1.82, 15.9) | 0.002 |
| Not reported | – 0.107 (0.499) | 0.899 (0.326, 2.34) | 0.830 |
| Baseline CMV DNA level intermediate/high | – 1.01 (0.489) | 0.363 (0.129, 0.910) | 0.030 |
| Prior use of CMV prophylaxis | – 0.818 (0.409) | 0.441 (0.191, 0.969) | 0.041 |

Reference subject did not develop treatment-emergent maribavir resistance at any time, had CD8+CD69+ cell count of <0.5%, low CMV DNA level at baseline, and did not use medication for CMV prophylaxis.

AUC_ss_, area under the plasma concentration–time curve at steady state on the last day of exposure; CI, confidence interval; CMV, cytomegalovirus; OR, odds ratio; SE, standard error

**Supplementary Fig. S1.1** Maribavir exposure in the exposure-efficacy population by key secondary endpoint: confirmed CMV clearance and CMV infection symptom control at week 8, maintained through week 16


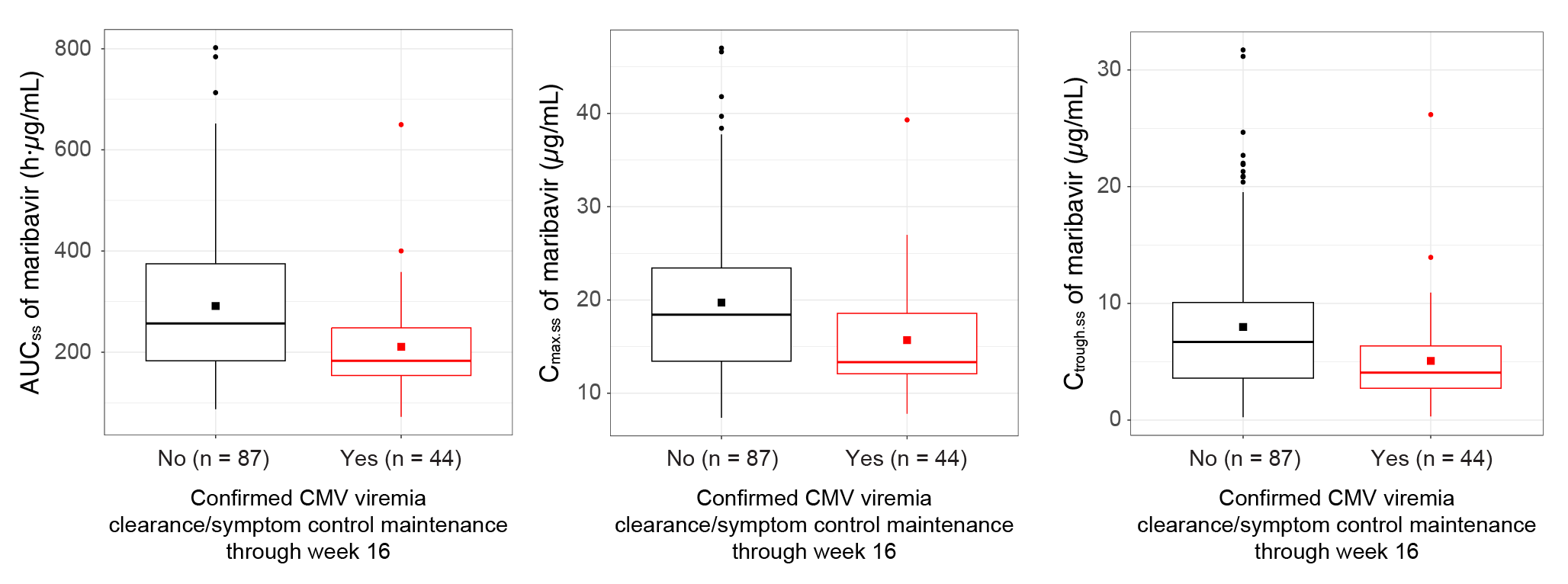


The lower and upper hinges correspond to the 25th and 75th percentiles, respectively. The upper and lower whiskers extend from the hinge to the largest or smallest value, respectively, no further than 1.5 × IQR from the hinge. Square symbols represent the arithmetic mean, circles represent outliers (i.e., data beyond the end of the whiskers).

AUC_ss_, area under the plasma concentration-time curve at steady state on the last day of exposure; C_max.ss_, maximum concentration of maribavir at steady state on the last day of exposure; CMV, cytomegalovirus; C_trough.ss_, minimum concentration of maribavir at steady state on the last day of exposure; IQR, interquartile range

**Supplementary Fig. S1.2** Probability of achieving key secondary endpoint: confirmed CMV clearance of plasma CMV DNA and CMV infection symptom control at week 8 followed by maintenance to week 16, as a function of area under the plasma concentration-time curve at steady state on the last day of exposure


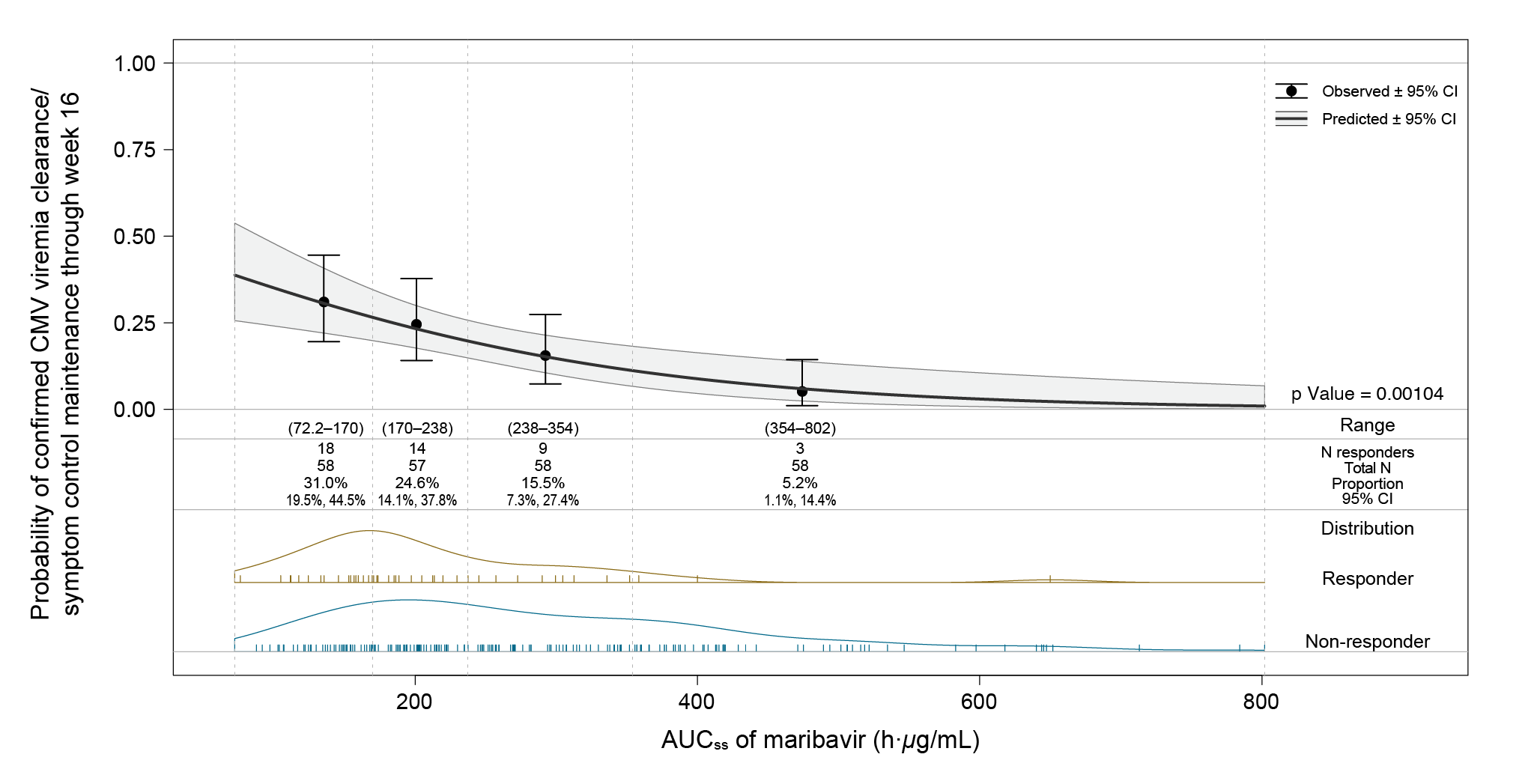


AUC_ss_, area under the plasma concentration-time curve at steady state on the last day of exposure; CI, confidence interval; CMV, cytomegalovirus; OR, odds ratio

**Supplementary Fig. S1.3** Probability of taste disturbance as a function of average maribavir exposure on each study day


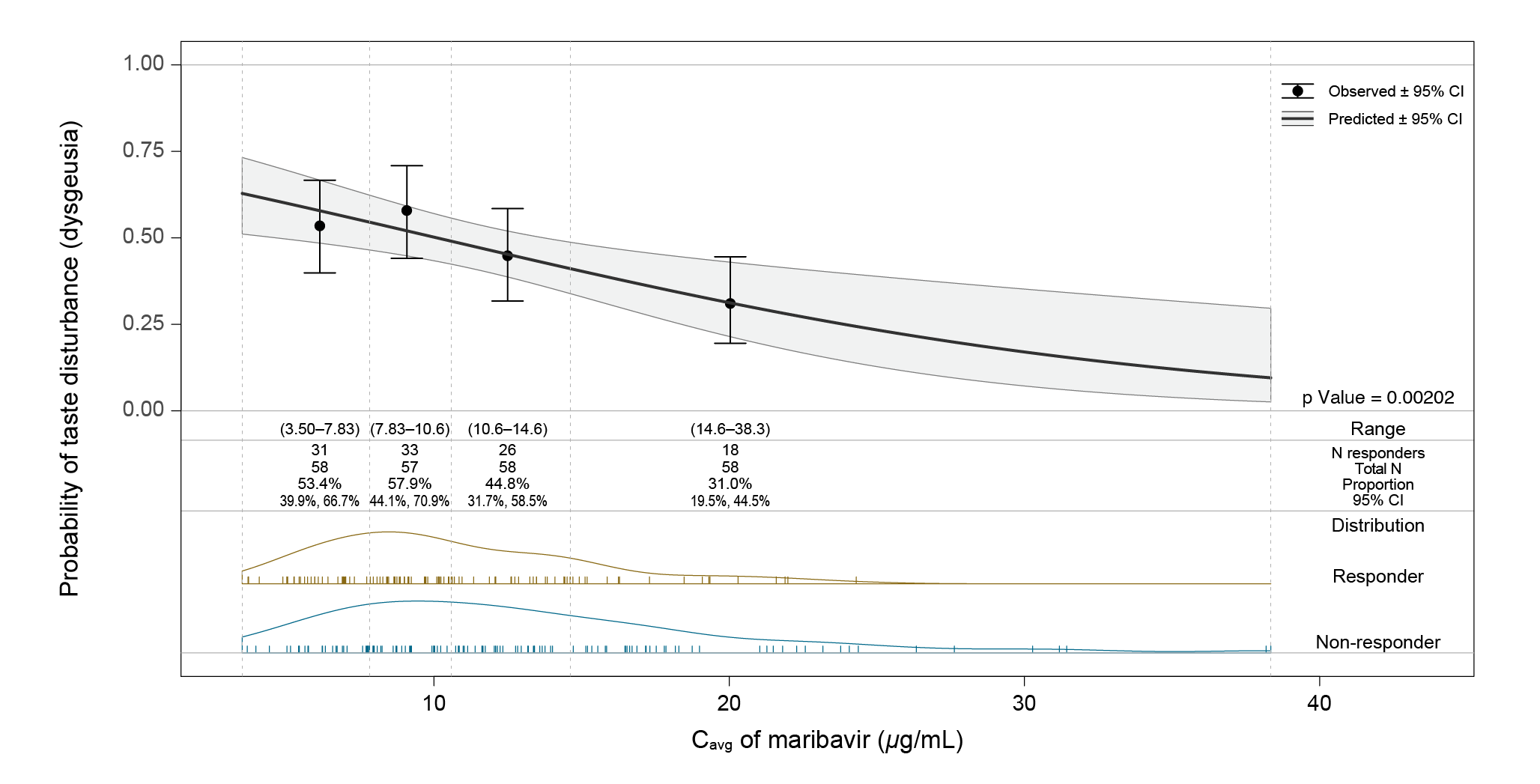


C_avg_, average maribavir exposure on each study day; CI, confidence intervals; N, number of patients

**Supplementary Fig. S1.4**  Probability of fatigue as a function of maximum concentration of maribavir at steady state on the last day of exposure.


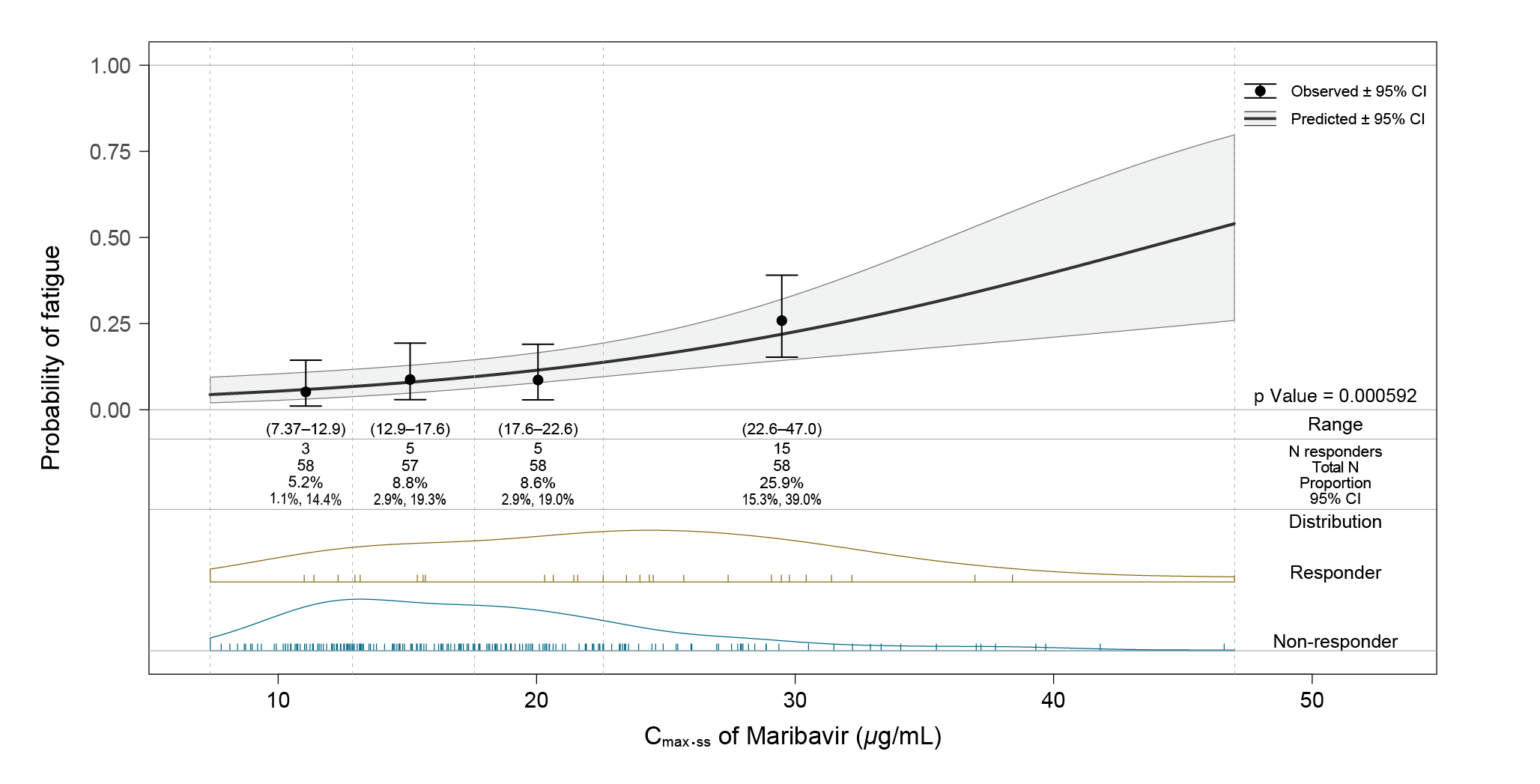


CI, confidence intervals; C_max.ss_, maximum concentration of maribavir at steady state on the last day of exposure; N, number of patients

**Supplementary Fig. S1.5** Probability of serious adverse events as a function of area under the maribavir plasma concentration-time curve at steady state on the last day of exposure


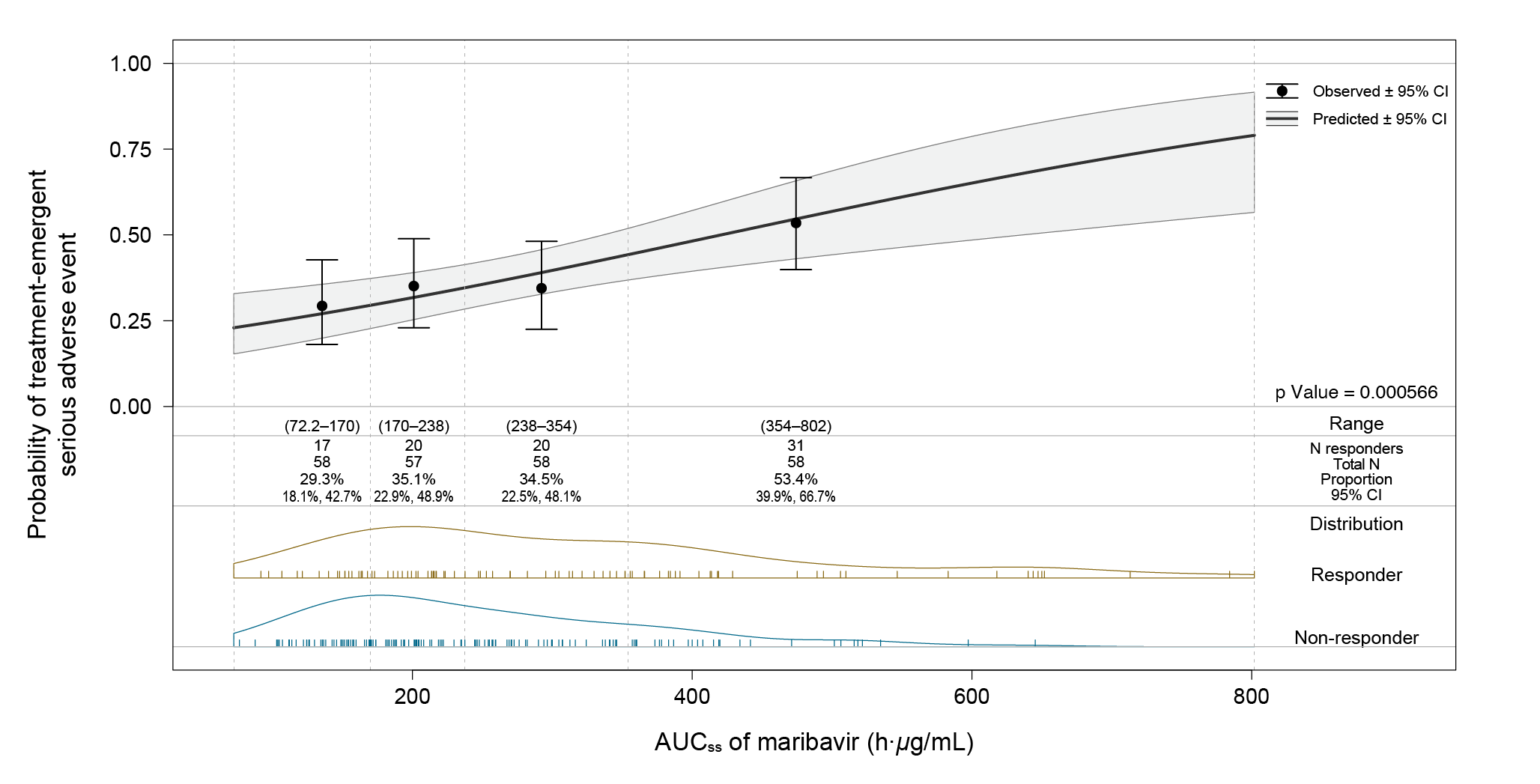


AUC_ss_, area under the plasma concentration–time curve at steady state on the last day of exposure; CI, confidence interval; N, number of patients
